# One Health Assessment of *Bacillus anthracis* Incidence and Detection in Anthrax-Endemic Areas of Pakistan

**DOI:** 10.3390/microorganisms11102462

**Published:** 2023-09-30

**Authors:** Nageen Sardar, Muhammad Waqar Aziz, Nadia Mukhtar, Tahir Yaqub, Aftab Ahmad Anjum, Maryam Javed, Muhammad Adnan Ashraf, Rabia Tanvir, Alan J. Wolfe, Daniel S. Schabacker, Sara Forrester, Mark Khemmani, Amin A. Aqel, Muhammad Akib Warraich, Muhammad Zubair Shabbir

**Affiliations:** 1 Institute of Microbiology, University of Veterinary and Animal Sciences, Lahore 54000, Pakistan; nageen.sardar@uvas.edu.pk (N.S.); maryam.javed@uvas.edu.pk (M.J.); adnan.ashraf@uvas.edu.pk (M.A.A.); rabia.tanvir@uvas.edu.pk (R.T.); shabbirmz@uvas.edu.pk (M.Z.S.); 2 Department of Microbiology, University of Jhang, Jhang 35200, Pakistan; 3 Department of Microbiology, The Islamia University of Bahawalpur, Bahawalpur 63100, Pakistan; 4 Department of Microbiology and Immunology, Loyola University Chicago, Chicago, IL 60660, USA; awolfe@luc.edu (A.J.W.);; 5 Argonne National Laboratory, Lemont, IL 60439, USA; 6 Faculty of Medicine, Mutah University, Al-Karak 61710, Jordan; aminaq@mutah.edu.jo; 7 Department of Marketing, Rennes School of Business, CS 76522, 2 Rue Robert d’Arbrissel, 35065 Rennes Cedex, France; muhammad-akib.warraich@rennes-sb.com

**Keywords:** *Bacillus anthracis*, human seroprevalance, environmental factors, emerging zoonotic disease, endemic

## Abstract

Anthrax, a severe zoonotic disease, is infrequently reported in anthrax-endemic regions of Pakistan. Despite clinical reports indicating its presence, particularly cutaneous anthrax, there is insufficient laboratory evidence regarding disease occurrence and environmental persistence. The present study aimed to confirm *Bacillus anthracis* presence, accountable for animal mortality and human infection, while exploring environmental transmission factors. Between March 2019 and July 2021, a total of 19 outbreaks were documented. Of these, 11 affected sheep/goats in Zhob district and 8 affected cattle/sheep in Bajour Agency. Clinical signs suggestive of *Bacillus anthracis* outbreak were observed in 11 animals. Blood and swab samples were collected for confirmation. The study followed a One Health approach, analyzing animal, environmental (soil/plant), and human samples. Of the 19 outbreaks, 11 were confirmed positive for anthrax based on growth characteristics, colony morphology, and PCR. Soil and plant root samples from the outbreak areas were collected and analyzed microscopically and molecularly. Cutaneous anthrax was observed in six humans, and swab samples were taken from the lesions. Human serum samples (*n* = 156) were tested for IgG antibodies against PA toxin and quantitative analysis of anthrax toxin receptor 1 (ANTXR1). *Bacillus anthracis* was detected in 65 out of 570 (11.40%) soil samples and 19 out of 190 (10%) plant root samples from the outbreak areas. Four out of six human samples from cutaneous anthrax lesions tested positive for *Bacillus anthracis*. Human anthrax seroprevalence was found to be 11% and 9% in two districts, with the highest rates among butchers and meat consumers. The highest ANTXR1 levels were observed in butchers, followed by meat consumers, farm employees, meat vendors, veterinarians, and farm owners. These findings highlight the persistence of anthrax in the region and emphasize the potential public health risks.

## 1. Introduction

Anthrax is a lethal zoonotic disease caused by bacterium *Bacillus anthracis* [1]. Domestic herbivores are most affected by the illness, resulting in large economic losses owing to animal death [2]. Humans mainly contract the infection via diseased animals or their products, or from exposure during clinical and agricultural practices [3]. Anthrax in humans presents in four clinical forms: cutaneous, gastrointestinal, pulmonary, and injectional [4], with the cutaneous form accounting for the vast majority of infections globally [5,6]. The disease’s clinical appearance in susceptible herbivores is characterized by abrupt death with or without bleeding from natural orifices, subcutaneous hemorrhages, fever, dyspnea, agitation, and convulsions, followed by splenomegaly, failure of blood coagulation, and lack of rigor mortis [7,8,9].

Anthrax is found all over the world and is endemic in various regions of South Europe, Asia, Africa, North and South America, and Australia [10]. Previous characterizations of worldwide diversity in anthrax endemism and outbreak intensity were restricted to coarse scales, and the illness was deemed a neglected disease since it received little public health attention [11]. Anthrax is considered endemic in several regions of Pakistan, notably in the provinces of Balochistan and Khyber Pakhtunkhwa [12]. For many years, anthrax outbreaks have been recorded and under-reported in Pakistan, usually as occasional outbreaks in numerous endemic hotspot locations impacting humans and pastoral animals [13]. In these endemic areas of the country, farmers observed that grazing animals are more prone to get anthrax infection compared to non-grazing animals (having stall feeding). Black eschars are also observed in humans in these endemic areas. However, there is a scarcity of thorough scientific data on the disease’s epidemiology, transmission dynamics, and ecological factors involved in disease transmission.

Livelihoods of communities dependent on livestock are vulnerable to the health status of their animals [14]. However, when faced with sick animals, the need to use or consume them creates a dilemma, leading to increased risk of anthrax exposure [15]. This, in turn, leads to the release of billions of *Bacillus anthracis* spores into the surrounding environment. The global increase in domestic animal populations has resulted in densely populated livestock regions, leading to persistent anthrax hotspots [16].

*Bacillus anthracis* spores can survive in soil for decades due to their strong tolerance of environmental stimuli [17]. Cattle and sheep can become infected with anthrax by grazing on polluted pastures or drinking from contaminated water sources [8,18]. Local meteorological conditions can influence the chance of animals coming into contact with *B. anthracis* spores, either directly or indirectly. This can be caused by grazing closer to the soil during dry seasons when grasses are short or sparse, as well as herd relocation to sheltered regions to save animals when water becomes limited [19]. The overall health of hosts can also influence their resistance to infection. Plant roots are also important in the spread of anthrax to animals and humans. *B. anthracis* spores can cling to the roots of some plant species in anthrax-endemic regions, where they can be consumed by grazing herbivorous animals [20]. While the exact mechanisms of anthrax spore adhesion to plant roots are unknown, root exudates, soil pH, and nutrient availability may all play a role [21]. Therefore, the development of control strategies requires a thorough understanding of the link between plant species, soil conditions, and anthrax transmission.

Despite the global dispersion of anthrax and its impact on animals and health in Pakistan’s anthrax-endemic regions, there is still a lack of understanding of the ecological and environmental elements that contribute to *B. anthracis* persistence and transmission. Averting outbreaks and highlighting the need for enhanced anthrax surveillance and management approaches are required. Therefore, the purpose of this study was to assess the possible contributions of several environmental elements, such as soil and plant roots, to the transmission of anthrax to herbivorous animals and humans in Pakistan’s anthrax-endemic areas. The investigation also sought to investigate the potential of molecular epidemiology methods for strengthening anthrax surveillance and control strategies.

## 2. Methodology

### 2.1. Study Areas and Sampling Strategy

Two anthrax-endemic districts (Zhob district of Balochistan province and Bajour Agency district of Khyber Pakhtunkhwa province) were selected for sampling, based on the clinical incidence of animal anthrax outbreaks in the past five years. Samples were collected between March 2019 and July 2021 during which 19 anthrax-suspected outbreaks in animals were reported. Of these outbreaks, 11 occurred in sheep/goats in Zhob district and 8 in Bajour Agency (5 in cattle and 3 in sheep). In Bajour district, cutaneous anthrax cases were also observed during this period.

### 2.2. Case Definition and Inclusion Criteria for Study

Animals affected by an anthrax outbreak, characterized by sudden mortality with or without oozing of blood from natural orifices and blotches, were included in the sample collection. In addition to the animal samples, environmental samples, including plant roots and soil, were collected from the sites of these anthrax outbreaks.

Individuals with black eschar or butcher’s warts on their skin, as well as those who had had direct or indirect contact with these individuals or with suspected anthrax-infected animal carcasses, were included in the blood sampling to confirm the seroevidence of anthrax exposure.

### 2.3. Animal Swab/Blood Samples

A total of 380 swab/blood samples, representing 19 outbreaks, were collected from natural orifices of anthrax-suspected carcasses having sudden mortality as shown in Table 1. In 11 outbreaks, blood was oozing from natural orifices. A total of 20 samples from each outbreak were collected which included swab/blood samples from the nasal cavity, oral cavity, rectum and blood from the jugular vein. The swabs were then pooled into sterile transport tubes and transported to the laboratory at 4 °C in ice boxes.

### 2.4. Environmental Samples

**Figure 1:**
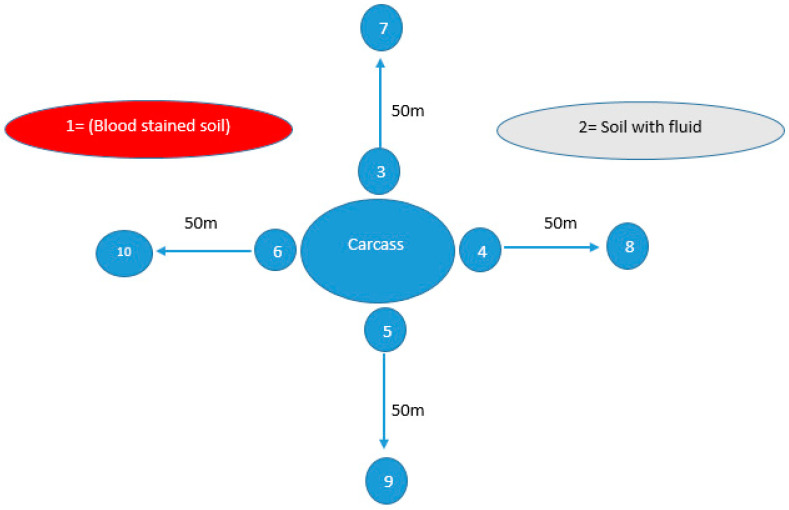
Soil sampling sites from areas suspected of anthrax outbreaks, illustrating ten distinct locations from which soil samples were collected within a single outbreak. Site 1, characterized by blood-stained soil, and Site 2, featuring soil contaminated with bodily fluids, were identified as locations where animal carcasses had either expired or were disposed of. Meanwhile, Sites 3, 4, 5, and 6 were sites surrounding the carcass in all four directions (east, west, south, north), while Sites 7, 8, 9, and 10 were located 50 m away in all these four directions.

A total of 190 plant root samples were collected from areas suspected to have had anthrax outbreaks in both districts (10 from each outbreak) as shown in Table 1. Plant root sampling sites were systematically chosen within a 10–15 m radius according to the availability of plants in areas where animals were housed, expired, or underwent disposal. For the soil sampling, 570 samples from the same outbreak sites were collected as shown in Table 1. From each outbreak, soil samples were collected from different sites including blood-stained soil, soil with non-hemorrhagic fluid, sites around the carcass and sites 50 m away from the carcass (as negative control) in all four directions as shown in Figure 1. Approximately 10 g of soil was collected from a depth of 4–6 inches from every site using a sterile spoon within 24–48 h following the expiration of the animal. These collected soil samples were placed into sterile zip-lock bags before being transported to the laboratory in a cooler at 4 °C.

### 2.5. Human Blood/Swab Samples

In the Bajour Agency and Zhob district regions, human blood samples were collected from individuals who had come into close contact with animals suspected to be carrying the anthrax disease. In Bajour Agency, a total of 112 blood samples were collected, out of which six individuals were observed to have symptoms indicative of cutaneous anthrax. Swab samples (*n* = 18, 3 swabs from each individual) from these cutaneous anthrax lesions were also collected for the isolation of *Bacillus anthracis*. In contrast, in Zhob district, 44 blood samples were collected, but none were found to have symptoms of cutaneous anthrax as shown in Table 1. From both districts, the samples were collected from individuals who had come into contact with potentially infected carcasses, as well as their families, farm owners, workers, butchers, and veterinarians. To ensure the samples were not contaminated, sterile blood collection tubes were used, and the samples were kept cool during transportation by storing them in an ice box at 4 °C.

### 2.6. Sample Processing

For sample processing, the following methods were adopted:

#### 2.6.1. Microbiological and Molecular Testing of Animal Swab Samples

To confirm the presence of *Bacillus anthracis* in suspected animal swab samples, microbiological testing was conducted in the biosafety level 3 laboratory (BSL3) at the Institute of Microbiology, University of Veterinary and Animal Sciences, Lahore. For the testing of hemolysis, samples were cultured on blood agar, which is a differential medium. Samples were also cultured on Polymyxin-lysozyme-EDTA-thallous acetate (PLET) agar, a selective medium specifically used for *B. anthracis*. In addition, Gram staining and the Schaeffer–Fulton method, utilizing Malachite Green for spore staining, were employed to identify Gram-positive rod-shaped bacteria characterized by the presence of green-colored spores in elongated chains. Motility testing was conducted using the hanging drop method [22]. 

#### 2.6.2. Bacterial Genomic DNA Extraction and Polymerase Chain Reaction

For molecular confirmation, suspected *B. anthracis* colonies on both PLET agar and blood agar were used for DNA extraction using a commercially available kit (QIAamp^®^ DNA Mini Kit). To confirm the presence of the bacterium, the polymerase chain reaction (PCR) was performed using the already reported primers for the plasmid-encoded capsular (CAP) gene, lethal factor (LF), edema factor (EF), protective antigen (PA), and bacterial chromosome [23]. To distinguish between vaccine strains and field strains, the final verdict was based on the presence of all five genes encoded by both plasmids (pX01, pX02), with particular emphasis on the capsular gene, as vaccine strains lack this specific gene due to the absence of a capsule.

#### 2.6.3. Turner Method for Plant Root Samples Processing

Plant root samples were processed using the Turner method [24]. Briefly, this method involved placing 5 g of plant roots into a sterilized 50 mL Falcon tube containing 45 mL of 0.1% sodium pyrophosphate solution. The tube was vortexed vigorously for 15–20 min to dislodge the spores from the plant roots. The solution was then centrifuged at 1600 rpm for 2 min to settle the debris, and the supernatant was centrifuged again at 6000 rpm for 15 min to obtain a pellet. The pellet was suspended in 5 mL of sodium pyrophosphate solution for microscopic and molecular analysis. 

#### 2.6.4. GABRI Method for Soil Sample Processing

The GABRI method was used to process soil samples for the detection of anthrax spores as described in a previous study [25]. Approximately 10 g of soil collected from the suspected anthrax outbreak areas were added to a sterile 50 mL Falcon tube. A GABRI buffer solution was added to the Falcon tube, and the tube was shaken vigorously for 30 min to release the spores from the soil particles. The solution was then centrifuged at 3000 rpm for 10 min and the supernatant solution was collected in a sterile Falcon tube. To eliminate the vegetative form of bacteria, the solutions obtained from the washing of plant roots and soil were subjected to a temperature of 65 °C for a duration of 60 min. Subsequently, the samples were cultured on PLET and blood agar media and a PCR of the suspected samples was also performed, following the previously described procedure.

#### 2.6.5. ELISA of Human Samples for Antibody Detection

The qualitative detection of antibodies against the protective antigen (PA) was carried out on serum samples through enzyme-linked immunosorbent assay [24] utilizing a commercially available kit known as the Human Anthrax Protective Antigen IgG (anti-PA-IgG) ELISA Kit (abx 055843). This assay operates on the principle of indirect enzyme-linked immunosorbent assay technology. Briefly, serum samples were initially diluted at a 1:5 ratio using the provided kit’s dilution buffer. Control samples, including one blank, two positive, and two negative controls as per kit instructions, were handled without dilution. Subsequently, 50 µL of these diluted samples and controls were loaded into wells and incubated for 30 min at 37 °C, followed by five washes with the kit’s washing buffer (1:20 dilution). Afterward, 100 µL of horseradish peroxidase enzyme conjugate was added, and another 30-min incubation at 37 °C was performed with subsequent washing. Then, 50 µL of substrates A and B were added to the wells and incubated for 15 min at 37 °C. Finally, 50 µL of stop solution was added, and the optical density at 450 nm was measured. The cut-off value, established by adding 0.15 to the mean OD of the negative control as per kit instructions, was used to classify samples with an OD value greater than or equal to the kit’s specified value as positive.

#### 2.6.6. ELISA of Human Samples for Anthrax Toxin Receptor 1 Quantification

The quantitation of ANTXR1 (Anthrax Toxin Receptor 1) was performed on serum samples using a commercially available kit, specifically the Human ANTXR1 ELISA kit (Fine Test EH 13997, Wuhan, China). This kit operates on the principle of sandwich enzyme-linked immune sorbent assay technology.

#### 2.6.7. Microscopic and Molecular Confirmation of Bacillus Anthracis in Human

For the microscopic confirmation of *Bacillus anthracis* from cutaneous anthrax lesions, swabs were cultured firstly in the enriched media (nutrient broth) and later on the differential media (blood agar) and selective media (PLET agar) as described earlier. Gram staining, the Schaeffer–Fulton method for spore staining, and the motility test using the hanging drop method were utilized to differentiate Gram-positive rod-shaped bacteria, distinguishing between those exhibiting green-colored spores arranged in elongated chains and non-motile bacterial strains. The suspected cultures were also tested molecularly via PCR.

#### 2.6.8. Assessment of Personal Demographics and Contact with Anthrax Suspected Animals

A standardized questionnaire was used to determine possible risk factors for *Bacillus anthracis* exposure. The questionnaire gathered personal demographic information such as gender, age, and occupation, as well as behavioral data on interactions with farm animals. Furthermore, we collected information on the date of symptom onset, history of slaughtering diseased animals, consumption of meat from diseased animals, disposal of suspected anthrax carcasses, occupation (farm owners/workers or butchers), presence of cutaneous lesions, and slaughtering practices. The collection of data included information on a history of direct or indirect contact with meat, excluding the activities of slaughtering and butchering.

#### 2.6.9. Statistical Analysis

The data regarding anthrax seroprevalence and Human Anthrax Toxin Receptor 1 concentrations among different occupational groups was analyzed using descriptive statistics and Fisher’s exact test. The adjusted Odds ratios (95% Confidence Interval) regarding seroprevalence among different occupational groups was calculated using the binary logistic regression in RStudio version 2023.06.1 (Build 524).

## 3. Results

### 3.1. Microbiological and Molecular Testing of Animal Swab Samples

On the basis of Gram staining, 127 samples exhibited the long chains of Gram-positive rods with square ends. Furthermore, of these 127 staining-positive samples, 82 showed suspected colonies of *B. anthracis* on selective media (PLET agar), characterized by grayish, circular, and dome-shaped colonies. Subsequently, these suspected samples were sub-cultured on blood agar to estimate non-hemolytic colonies for further confirmation. Finally, 66 samples corresponding with 11 outbreaks exhibited non-hemolytic, large white to grey colonies with irregular margins resembling Medusa’s head. These 66 samples were used for the molecular confirmation of *B. anthracis.* PCR of these 66 samples revealed that 44 samples were positive for all five genes including the capsular gene of *B. anthracis* as shown in Table 2.

### 3.2. Microscopic and Molecular Testing of Plant Root Samples

In the Gram staining procedure, 95 samples were identified as containing Gram-positive rods. 81 samples were characterized as “suspected” based on their possession of long chain rods with square ends. Among these 81 samples, 59 were confirmed to contain spores upon further examination via spore staining. These 59 samples were subsequently cultured on both PLET and blood agar. After this process, a total of 42 samples were identified as “suspected” based on their colony characteristics. In the molecular testing phase, genomic DNA was extracted from all 42 samples that were initially suspected of containing spores based on staining. PCR was then conducted, which confirmed the presence of the *Bacillus anthracis* genes in 19 out of the 42 suspected samples as shown in Table 2.

### 3.3. Microscopic and Molecular Testing of Soil Samples

In the Gram staining procedure, 253 of these 380 samples were characterized as “suspected” based on their possession of long chain rods with square ends. Among these 253 samples, 223 were confirmed to contain spores upon further examination via spore staining. These 223 samples were subsequently cultured on both PLET and blood agar. After this process, a total of 162 samples were identified as “suspected” based on their colony characteristics. In the molecular testing phase, genomic DNA was extracted from all 162 samples that were initially suspected of containing spores based on staining. Polymerase chain reaction was then conducted, which confirmed the presence of the *Bacillus anthracis* genes in 65 of the 162 suspected samples as shown in Table 2.

### 3.4. Microscopic and Molecular Testing of Human Swab Samples

**Figure 2:**
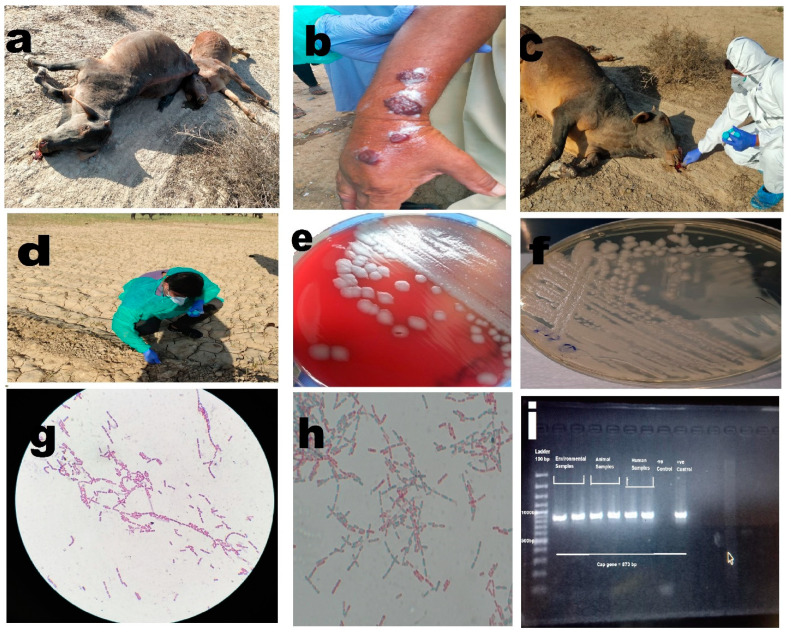
(**a**) Anthrax outbreak in animals, illustrating cases of infection among animals. (**b**) Cutaneous anthrax case in a human, depicting an individual affected by cutaneous anthrax. (**c**) Collection of soil samples around the carcass, highlighting the process of collecting soil samples in the vicinity of animal carcasses. (**d**) Collection of plant root samples from infected sites, showing the collection of plant root samples from areas affected by anthrax. (**e**) Non-hemolytic growth of *Bacillus anthracis* on blood agar, displaying the characteristic non-hemolytic growth pattern of *Bacillus anthracis* on blood agar medium. (**f**) Growth of *Bacillus anthracis* on PLET agar, illustrating the growth of *Bacillus anthracis* on PLET agar medium. (**g**) *Bacillus anthracis* showing Gram-positive rods in long chains, visualizing the Gram-positive rod-shaped morphology of *Bacillus anthracis* arranged in elongated chains. (**h**) *Bacillus anthracis* with spores, depicting the presence of spores in *Bacillus anthracis*. (**i**) PCR bands for *Bacillus anthracis* capsular gene, showcasing the PCR results demonstrating specific bands corresponding to the capsular gene of *Bacillus anthracis*.

Of the six individuals suspected of having cutaneous anthrax, specific growth on selective and differential media, microscopic confirmation, and molecular confirmation yielded positive results for four cases from 18 swab samples from six individuals as shown in Table 2 and Figure 2 All of these individuals had histories of slaughtering animals.

### 3.5. ELISA of Human Samples for IgG Antibody Detection

**Figure 3:**
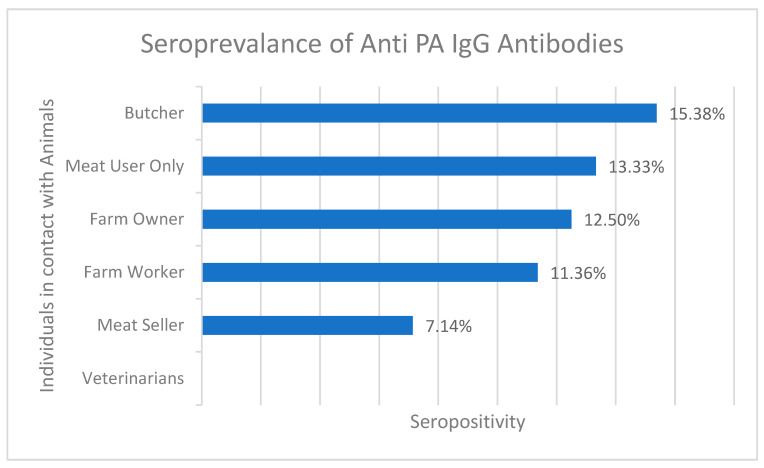
Percentage seroprevalence of anti-PA IgG antibodies in different individuals who were in contact with infected animals.

Based on the results of the anti-PA ELISA test, it was found that IgG antibodies against anthrax were detected in 11 of 100 serum samples from District Bajaur KPK. The overall seroprevalence sample percentage from Bajour district was calculated to be 11% (*n*= 11/100), indicating the presence of anthrax in the area. The highest seroprevalence was observed among butchers (15.38%, 2/13) and meat users (13.33%, 2/15), followed by farm owners (12.5%, 1/8), farm workers (11.36%, 5/44), and meat sellers (7.14%, 1/14). Interestingly, none of the samples collected from veterinarians (*n* = 6) tested positive for anthrax antibodies. In the Zhob district, IgG antibodies were detected in 4 of 44 (9%) human samples as shown in Figure 3 and Supplementary Table S1. These findings suggest that the anthrax organism is still present in the field and poses a potential threat to public health.

### 3.6. ELISA of Human Samples for Anthrax Toxin Receptor 1 Quantification

**Figure 4:**
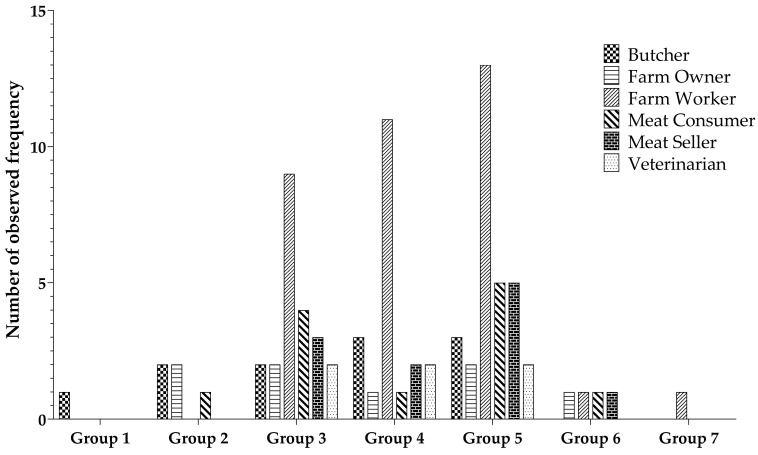
Distribution of individual sample counts across varied groups based on ANTXR1 concentration levels.

The ANTXR1 quantitative ELISA assay was used to determine the level of ANTXR1 in various professions associated with animal contact. Participants primarily consisted of farm workers (44%), farm owners (8%), butchers (13%), meat sellers (14%), and veterinarians (6%). Participants’ samples were categorized into seven groups according to their ANTXR1 concentration levels. Group 1 exhibited the highest concentration, while group 7 had the lowest concentration, with a gradual decrease in concentration from group 1 to group 7. Notably, group 5 contained the largest number of samples, with subsequent placements held by group 4 and group 3, as visually depicted in Figure 4. There is a non-significant difference regarding the observed frequency among the different occupational groups.

## 4. Discussion

The prevalence and impact of anthrax outbreaks, both on animals and humans, underscore the urgency of understanding its transmission and devising effective control strategies. This study delves into the epidemiology of anthrax in two anthrax-endemic districts, Zhob and Bajour Agency, selected due to their historical animal anthrax incidence. The pressing need for such investigation lies in the potential public health threats posed by anthrax, a zoonotic disease that can result in severe economic losses and human casualties. By elucidating the spatiotemporal patterns of outbreaks and employing a multidisciplinary approach involving microbiological, molecular, and serological techniques, the study aims to shed light on the complex interactions between infected animals, the environment, and human populations. Understanding these interactions is pivotal for devising targeted intervention strategies and enhancing overall preparedness to mitigate the impact of anthrax outbreaks on both animal and human health.

The study design employs a comprehensive methodology to investigate the multifaceted dynamics of anthrax prevalence. These selected districts provide a pertinent context for understanding the prevalence and characteristics of the disease. Extending from March 2019 to July 2021, the data collection period coincided with 19 anthrax-suspected outbreaks, which included 11 in sheep/goats in Zhob district and 8 in cattle/sheep in Bajour Agency. The study’s inclusiveness is evident through the integration of animal, environmental, and human samples. Animal swab/blood, plant root, soil, and human swab/blood samples were meticulously collected. The multidimensional analysis encompassed microbiological testing, molecular techniques like PCR, and serological assays [22]. This methodological approach offers a nuanced understanding of the disease’s occurrence, transmission pathways, and potential reservoirs, thus facilitating informed decision-making for anthrax management and control. The study’s findings provide information on the frequency of anthrax in the study region and emphasize the possible public health danger presented by the anthrax bacterium.

Microbiological testing of animal swab/blood samples revealed that 66 out of 380 samples were positive based on Gram and spore staining, and samples exhibited non-hemolytic, large white to grey colonies with irregular margins resembling Medusa heads, indicating the presence of *B. anthracis*. These findings are consistent with earlier research that has used Gram staining and spore staining to detect *B. anthracis* in animal samples [26]. Previous research has also described the use of selective medium for *B. anthracis* isolation, such as PLET agar [27]. The presence of *B. anthracis* in animal swab samples is especially concerning since it implies that the anthrax organism is still prevalent in the wild and constitutes a risk to animal and human health. The virulence of *B. anthracis* hinges on the presence of two plasmids, specifically pX01 and pX02. Plasmid pX01 carries the genetic information responsible for encoding edema factor (EF), lethal factor (LF), and protective antigen (PA), while pX02 encodes for the production of the bacterial capsule [28]. Remarkably, the PCR-based confirmation of the five genes further substantiated the prevalence of *B. anthracis* in these outbreaks. This aligns with previous studies that also underscored the importance of animals as a reservoir for the pathogen [29].

The investigation of environmental samples, including plant roots and soil, yielded noteworthy results. Microscopic and molecular testing of plant root samples revealed the presence of spores in 19/190 (10%) samples, suggesting a potential route for spore dissemination through contaminated vegetation. This conclusion is consistent with earlier research that found *B. anthracis* in plant materials such as soil and grass [30]. The discovery of *B. anthracis* in plant root samples emphasizes the potential dangers of anthrax exposure in the environment. Similarly, the study’s analysis of soil samples highlighted the presence of anthrax spores in 65/570 (11.4%) samples, reflecting the environmental persistence of the pathogen. These findings corroborate previous research that emphasized the significance of soil as a medium for spore survival and dissemination [30,31].

The human aspect of anthrax transmission was also explored comprehensively. Serological analysis of human blood samples demonstrated the presence of IgG antibodies against the anthrax protective antigen, indicating prior exposure. Human anthrax seroprevalence was found to be 11% in Bajour and 9% in Zhob district’s samples. The observed seroprevalence rates varied across occupational groups, with butchers (15.38%) and meat consumers (13.33%) showing higher rates. This parallels previous studies that identified occupational exposure as a significant risk factor for anthrax transmission in individuals who interact closely with animals and animal products [32,33].

The presence of serum ANTXR1 levels has been identified as a potential indicator for assessing the correlation between its existence and the occurrence of anthrax infection or exposure [34]. In cases of anthrax infection, the binding of anthrax toxin to cell-associated ANTXR1 can result in its release into the bloodstream, leading to detectable levels of soluble ANTXR1 in the serum [35]. Consequently, monitoring serum ANTXR1 levels holds promise as a biomarker for detecting anthrax exposure or infection. Our study aimed to investigate whether variations in exposure to anthrax spores or toxins could be reflected in serum ANTXR1 concentrations across different occupational groups in anthrax-endemic areas. In our study, we observed the highest concentrations of serum ANTXR1 among individuals with a history of slaughtering diseased animals and those who consumed meat from these sources. While the direct correlation between serum ANTXR1 levels and anthrax infection remains a subject of ongoing research, our findings contribute valuable data that can inform and guide future investigations in this area.

Interestingly, the present study also unveils cases of cutaneous anthrax within the human population of a tribal community situated in the Bajour district. These individuals are known to have engaged in the slaughtering of animals and have experienced recurrent outbreaks of animal anthrax. Our investigation revealed a distinct clustering of human anthrax cases in regions where animals had died due to anthrax-related causes. Furthermore, the presence of cutaneous anthrax lesions in individuals with direct contact underscores the disease’s potential for zoonotic transmission.

Various socio-cultural practices, including the slaughter of sick animals, consumption or handling of meat from infected animals, and improper disposal of dead carcasses in open environments, have significantly contributed to the transmission of anthrax in previous outbreaks documented in Africa and Southeast Asian countries [36]. The act of scavenging meat from carcasses for consumption is culturally acceptable among certain local tribes in the Zhob and Bajour districts, and this practice has been identified as a contributing factor to anthrax transmission. The convergence of low socioeconomic status and limited education within the tribal community, coupled with inadequate public health infrastructure, creates a confluence of risk factors conducive to the zoonotic transmission of anthrax to humans [37]. Enhancing the community’s awareness regarding potential risk factors for illness could potentially prevent future outbreaks.

In response to the outbreaks reported in the current study, efforts were made to organize health education camps aimed at sensitizing the community to behavioral changes that could help prevent anthrax. Community health workers conveyed simple yet crucial health messages to all villagers, emphasizing the importance of not handling sick animals without proper protection and the safe disposal of deceased animals through disinfection, among other practices.

Comparing these findings with previous research, the current study provides a more integrated understanding of anthrax transmission by combining microbiological, molecular, and serological techniques. The multi-sample analysis approach involving animal, environmental, and human specimens enhances the depth of insights into anthrax’s intricate dynamics. This study’s findings align with earlier investigations in highlighting the role of animals, contaminated soil, and occupational exposure in anthrax transmission. However, the study’s strength lies in its comprehensive and multidisciplinary approach, which contributes to a holistic understanding of anthrax epidemiology and offers valuable data for designing targeted intervention strategies to curb its impact on both animal and human populations.

While the study provides valuable insights into anthrax detection in endemic areas, there are certain limitations that warrant consideration. Firstly, the study’s retrospective design relies on historical outbreak data, which might not encompass all instances of anthrax outbreaks within the selected districts. This could potentially lead to an under-representation of sporadic or unreported cases. Secondly, the sample collection period, spanning from March 2019 to July 2021, might not fully capture seasonal variations and long-term trends in anthrax incidence. Additionally, the study’s focus on specific districts within a particular region might limit the generalizability of findings to broader geographical contexts with varying ecological and demographic factors. Furthermore, while the integrated approach involving microbiological, molecular, and serological techniques enhances the study’s robustness, certain challenges such as sample contamination and assay sensitivity could affect the accuracy of results. Lastly, the study primarily relies on cross-sectional data, limiting the ability to establish definitive causal relationships between exposure and infection. Despite these limitations, the study contributes significantly to the understanding of anthrax transmission pathways and offers a foundation for informed public health strategies in endemic regions.

In conclusion, this study provides a comprehensive investigation into the epidemiology of anthrax in anthrax-endemic districts, shedding light on its intricate transmission dynamics among animals, the environment, and human populations. The integration of microbiological, molecular, and serological approaches yields a nuanced understanding of the disease’s prevalence, distribution, and potential sources. The confirmed presence of *Bacillus anthracis* in animal carcasses and the environment underscores the ongoing threat posed by anthrax spores. Human seroprevalence data further highlight the zoonotic nature of anthrax and emphasize the occupational risk faced by those closely interacting with animals and animal products. While limitations such as retrospective design and regional specificity exist, this study’s multidisciplinary approach enhances the overall comprehension of anthrax transmission pathways. The findings not only align with previous research but also offer unique insights into the complex interplay between host, environment, and pathogen. Ultimately, this study serves as a valuable foundation for devising targeted intervention strategies aimed at minimizing the impact of anthrax outbreaks on both animal and human health in endemic regions. 

## Figures and Tables

**Table 1:** Details of suspected anthrax outbreak, including information on infected species, clinical history, and the quantity of collected samples.

Outbreak No.	Date DD/MM/YY	Location	Animal	Brief Clinical History	Collected Samples				
					Animal Blood/Swab	Soil	Plant Roots	Human Swabs	Human Blood
1	7 March 2019	Zhob	Sheep	Sudden mortality, blood oozing out and bloating	20	30	10	0	4
2	29 March 2019	Zhob	Sheep	Sudden mortality	20	30	10	0	4
3	21 April 2019	Bajour	Goat	Sudden mortality	20	30	10	0	15
4	8 June 2019	Zhob	Goat	Sudden mortality, blood oozing out and blotches	20	30	10	0	4
5	14 July 2019	Bajour	Goat	Sudden mortality, blood oozing out and blotches	20	30	10	3	15
6	5 August 2019	Zhob	Goat	Sudden mortality	20	30	10	0	4
7	12 October 2019	Zhob	Sheep	Sudden mortality	20	30	10	0	4
8	17 April 2019	Bajour	Sheep	Sudden mortality, blood oozing out and blotches	20	30	10	3	15
9	4 May 2020	Bajour	Cattle	Sudden mortality, blood oozing out and blotches	20	30	10	3	15
10	22 June 2020	Zhob	Sheep	Sudden mortality	20	30	10	0	4
11	13 July 2020	Bajour	Sheep	Sudden mortality	20	30	10	3	15
12	5 August 2020	Zhob	Sheep	Sudden mortality, blood oozing out and blotches	20	30	10	0	4
13	16 October 2020	Zhob	Goat	Sudden mortality	20	30	10	0	4
14	4 November 2020	Zhob	Sheep	Sudden mortality, blood oozing out and blotches	20	30	10	0	4
15	12 March 2021	Bajour	Cattle	Sudden mortality, blood oozing out and blotches	20	30	10	0	15
16	2 April 2021	Zhob	Sheep	Sudden mortality	20	30	10	0	4
17	18 June 2021	Zhob	Sheep	Sudden mortality, blood oozing out and blotches	20	30	10	0	4
18	23 June 2021	Bajour	Cattle	Sudden mortality, blood oozing out and blotches	20	30	10	3	15
19	19 July 2021	Bajour	Sheep	Sudden mortality, blood oozing out and blotches	20	30	10	3	15

**Table 2:** Microscopic, growth, and molecular confirmation results of collected samples from suspected anthrax outbreaks, along with conclusive outcomes indicating both positive and negative cases.

	Microscopic and Growth Results				Molecular Confirmation				Conclusion
	No. of Positive Samples				No. of Positive Samples				
Outbreak No.	Animal Blood/Swab Samples	Soil	Plant Roots	Human Swabs	Animal Blood/Swab Samples	Soil	Plant Roots	Human Swabs	
1	4	9	2	0	4	3	0	0	Positive
2	3	13	0	0	0	0	0	0	Negative
3	6	7	0	0	0	0	0	0	Negative
4	2	6	3	0	3	5	2	0	Positive
5	4	9	0	2	4	6	3	2	Positive
6	4	12	1	0	0	0	0	0	Negative
7	3	5	2	0	0	0	0	0	Negative
8	6	11	0	3	2	2	0	2	Positive
9	4	10	4	1	4	8	2	3	Positive
10	2	13	2	0	4	9	1	0	Positive
11	2	4	3	2	7	10	3	0	Positive
12	3	8	0	0	3	6	2	0	Positive
13	4	6	6	0	0	0	0	0	Negative
14	3	9	4	0	0	0	0	0	Negative
15	2	12	5	0	0	0	0	0	Negative
16	3	5	4	0	3	3	2	0	Positive
17	4	6	6	0	4	7	1	0	Positive
18	2	10	0	3	6	6	3	2	Positive
19	5	7	0	0	0	0	0	0	Negative
**Total**	**66**	**162**	**42**	**11**	**44**	**65**	**19**	**9**	

Bold indicates the total number of positive samples.

## Data Availability

All the data is available.

## Supplementary Materials

The following supporting information can be downloaded at: https://www.mdpi.com/article/10.3390/microorganisms11102462/s1, Table S1: Seroprevalence of Anti-PA IgG Antibodies in Various Individuals Exposed to Infected Animals: Descriptive Statistics on Percentage Levels.
