# Supplementary material for: One Health Assessment of Bacillus anthracis Incidence and Detection in Anthrax-Endemic Areas of Pakistan
Source: Microorganisms. 2023 Sep 30;11(10):2462. doi: 10.3390/microorganisms11102462 (PMC10609008; doi:10.3390/microorganisms11102462)
Supplement: Supplementary file 1 [file microorganisms-11-02462-s001.zip › microorganisms-2608675-supplementary.pdf]

microorganisms

MDPI

Table S1. Seroprevalence of Anti-PA IgG Antibodies in Various Individuals Exposed to Infected Animals: Descriptive Statistics on Percentage Levels.

Seroprevalence Among Various Occupational Groups					
Variables	Total Observed	Positive (%)	Odds Ratio	95% CI	*P*-Vlue
**Farm Worker**	44	5 (11.36%)	0.83	0.14 – 4.60	0.84
**Farm Owner**	8	1 (12.5%)	0.93	0.057 – 9.13	0.95
**Butcher**	13	2 (15.3%)	1.18	0.16 – 8.45	0.87
**Meat Seller**	14	1 (7.14%)	0.5	0.03 – 4.81	0.58
**Veterinarians**	6	0	0	0	-
**Meat Consumer**	15	2 (13.33%)	*		

* Indicates the parameter was used as reference to calculate the Adjusted Odds ratio.
